# Face familiarity revealed by fixational eye movements and fixation-related potentials in free viewing

**DOI:** 10.1038/s41598-022-24603-w

**Published:** 2022-11-23

**Authors:** Oren Kadosh, Yoram Bonneh

**Affiliations:** 1 School of Optometry and Vision Science, Faculty of Life Sciences, Bar-Ilan University, Ramat-Gan, Israel; 2 The Leslie and Susan Gonda Multidisciplinary Brain Research Center, Bar-Ilan University, Ramat-Gan, Israel

**Keywords:** Cognitive neuroscience, Visual system, Object vision, Neuroscience, Oculomotor system, Saccades

## Abstract

Event-related potentials (ERPs) and the oculomotor inhibition (OMI) in response to visual transients are known to be sensitive to stimulus properties, attention, and expectation. We have recently found that the OMI is also sensitive to face familiarity. In natural vision, stimulation of the visual cortex is generated primarily by saccades, and it has been recently suggested that fixation-related potentials (FRPs) share similar components with the ERPs. Here, we investigated whether FRPs and microsaccade inhibition (OMI) in free viewing are sensitive to face familiarity. Observers freely watched a slideshow of seven unfamiliar and one familiar facial images presented randomly for 4-s periods, with multiple images per identity. We measured the occipital fixation-related N1 relative to the P1 magnitude as well as the associated fixation-triggered OMI. We found that the average N1-P1 was significantly smaller and the OMI was shorter for the familiar face, compared with any of the seven unfamiliar faces. Moreover, the P1 was suppressed across saccades for the familiar but not for the unfamiliar faces. Our results highlight the sensitivity of the occipital FRPs to stimulus properties such as face familiarity and advance our understanding of the integration process across successive saccades in natural vision.

## Introduction

Traditional neurophysiological studies of object perception typically probe the visual system with flashed stimuli, to mimic the saccade-induced transients of natural vision, measuring event-related potentials (ERPs). More recently, fixation-related potentials (FRPs) have been used to study vision in more natural settings of free viewing, demonstrating the advantages as well as the limitations of this method^1^. In general, these studies show results that are consistent with the event-related measurements; however, none of these studies probed face familiarity. Unlike traditional ERPs that used briefly flashed visual transients presented at the observer’s central visual field, in natural settings the scene is scanned over time via saccades following a peripheral preview.

### Free viewing

Accumulating evidence from recent free-viewing studies suggests that the brain’s response following a saccade, termed Fixation-related Potentials (FRP), exhibits electrophysiological components very similar to ERPs. For example, the saccade-related occipital lambda response reflects the same information processing as the classic VEP P1^2^. Recent studies that examined the face-selective activity at the lateral temporo-occipital electrodes, N170^3^ found a resembling increased negativity for faces in free viewing conditions^4,5^. More classic findings were replicated in free viewing conditions, such as centro-parietal P300 elicited by target detection in a visual search^6^ and the N400 priming effect in natural reading^7,8^. Combining EEG and eye-tracking measurements to study face familiarity enabled us to cross examine eye movement and electrophysiological changes over time, which are influenced by habituation and prior knowledge.

### Oculomotor inhibition

Microsaccades (MS) are miniature saccades, with an average size of < 0.5 dva, generated by neural activity in the superior colliculus (SC)^9,10^. They occur during fixation, with a rate of one or two per second. Microsaccades, as well as saccades, are known to be inhibited momentarily (Oculomotor-Inhibition, OMI)^11–16^ by stimulus presentation with a later release latency affected by the stimulus properties, attention and expectation. Whereas stimulus saliency, such as high contrast, is known to shorten the inhibition^17^, prolonged inhibition was found in response to deviants^18^. Although most studies used flashed stimuli, we have recently found similar inhibitory patterns in free viewing in response to stimulus saliency^19^.

### Face familiarity

Faces are considered complex stimuli that are processed holistically in posterior brain regions including the occipital face area (OFA), the fusiform face area (FFA), and the posterior superior temporal sulcus (STS), which form a core system for encoding the visual appearance of faces^20^. This activity is modulated by top-down feedback from personal knowledge and emotional responses to alter the processing of familiar faces^21^. Face familiarity is of a major interest in research on concealed memories; the Concealed Information Test (CIT) is used for testing the familiarity of suspects to specific people and objects^22^. Our recent study found prolonged OMI at fixation for a masked familiar face^23^, which allowed us to reliably detect identity in a concealed information test^24^. Next, we will review in more detail the currently known oculomotor and the ERP measures of familiarity.

### Face familiarity using oculomotor measures

Most of the experiments examining ocular measures in response to face familiarity used flashed stimuli. Rosenzweig & Bonneh^23^ measured the oculomotor inhibition of both microsaccades and blinks in response to masked novel and universally learned^23^ or recently learned^24^ familiar faces, and found prolonged OMI for the familiar faces in passive viewing. However, few studies have focused on different aspects of gaze fixations; one study claimed that the first two fixations are critical for revealing familiarity^25^, and other studies suggested that fewer fixations and longer fixation durations are the key for familiarity^26,27^. In contrast, another study reported more fixations and longer fixation durations for familiar faces with a CIT paradigm^28^; however, when participants had to memorize faces, familiar faces initially attracted their gaze, but later triggered fewer fixations and shorter fixation durations^29^. Overall, the eye movement behavior in relation to face familiarity depended on the instructions given by the researcher.

### Face familiarity using EEG measurements

Previous electrophysiological familiarity studies measured the late event-related (ERP) response including the N250^30,31^ and the P300 contextual response. The face-sensitive N170 component, which reflects the structural encoding of faces prior to person identification^32,33^ was also tested; however, there were conflicting results. The majority of the studies did not find a familiarity effect^30,31,34,35^, R. N.^36^; nevertheless, a few studies found an effect showing either larger N170 magnitudes for familiar faces^37–41^ or smaller than for unfamiliar ones^42,43^.

### Current study motivation and novelty

To date, little or no research has focused on earlier visual responses regarding face familiarity. However, a few studies examined the occipital P1 and N1 components using unfamiliar faces as stimuli, suggesting an early coarse processing of faces prior to face identification^44,45^. Here, we studied the fixation-triggered early posterior components that reflect feature and structure visual processing, and the timing of microsaccades during fixation, as well as saccades, while participants freely viewed large images of both famous and unfamiliar faces presented for several seconds. We expected to find differences in the modulation over time, across successive saccades of the early occipital as well as the oculomotor responses by prior knowledge in the context of familiarity. Thus, the aim of this study was to investigate whether the FRP and OMI in free viewing are sensitive to face familiarity.

## Methods

### Participants

A total of sixteen observers were recruited for the experiment: eight females and eight males, aged 21–44. One participant was omitted from the data analysis due to the low quality of the data (more than 50% bad data). All participants had normal or corrected-to-normal vision and were naïve to the purpose of the study. The experiments were approved by the Bar-Ilan Internal Review Board (IRB) Ethics Committee. All participants gave written informed consent, and all the experiments were conducted according to the IRB guidelines.

### Apparatus

The study combines eye tracking and electrophysiology recordings in free viewing synchronized by a split trigger to both systems. A wireless 8-channel headset with dry electrodes (Cognionics) was used for the EEG recordings and the Eyelink 1000 plus (SR Research) for eye tracking, both with a sampling rate of 500 Hz. The EEG signal had a built-in reference using channels placed on both ears. Stimuli were displayed using the in-house integrative stimulus presentation and analysis tool (PSY), developed by Y.S. Bonneh, by a 100 Hz calibrated 24-in FHD LCD monitor (Eizo Foris fg2421), at 0.6 m distance. The experiment was administered in dim light. We used a 35 mm lens positioned 0.52 m from the participant’s stabilized head using a chin rest. All recordings were done binocularly, with analyses done on data from the left eye. A standard 9-point calibration was performed before each session.

### Saccade and microsaccade RT calculation

For the saccade detection, we used an algorithm introduced by Engbert and Kliegl^46^, which is based on eye movement velocity. Microsaccades were detected as movements exceeding 8 *SD* of the mean velocity in 2D velocity space, as in^23,47^. A velocity range of 8°/s–150°/s, an amplitude range of 0.08–1°, and a minimum duration of 9 ms were allowed for the microsaccades. We calculated the Warren Sarle’s bimodality coefficient (*BC*) of the saccade amplitude data, which is associated with the data skewness and kurtosis using a Matlab function by Hristo Zhivomirov^48^. *BC* has a range of 0–1, where values greater than ~ 0.555 (the value for the uniform distribution) indicate bimodal or multimodal data distributions^49^. Eye tracking epochs were extracted, triggered by saccade (> 1 *dva*) landing time, as in our previous study^19^, in a range of − 0.2 s to 0.8 s relative to the fixation onset with some overlap between epochs. This was taken into consideration when computing the microsaccade Reaction Time (msRT). The msRT was calculated for each epoch relative to the fixation onset in a predefined time window, as the latency of the first microsaccade in that window. The first fixation per trial was always ignored to avoid the flash effect on the OMI. The microsaccade RTs (msRT) were averaged across the epochs of each condition within observers and then averaged across observers, with error bars computed across observers on demeaned (within observer) data, with a correction factor (multiplied by √(n/(n − 1))). This method for computing the error bars allows a better representation of within-participant effects (Cousineau & Morey’s method^50^; see also Bonneh et al.^17^. The inter-saccade interval, termed the fixation duration, was calculated as the time interval between the current fixation onset and the next fixation onset, including only MS-free fixations.

### Fixation-related potentials—FRP

The EEG data were filtered using a 0.1 Hz high-pass and 30 Hz low-pass cutoffs. FRP epochs were created, as was done for the eye-tracking data, triggered by the saccade (> 1 *dva*) landing time in a range of − 0.1 s to 0.3 s*,* relative to the fixation onset to minimize overlapping data between epochs. The first fixation per trial was always ignored to avoid the flash effect. Since our EEG system had only eight channels and is not equipped with EOG electrodes, ICA and deconvolution methods for correcting ocular artifacts were not used. Instead, overlapping data points between proximal saccades were excluded on both epochs triggered by those saccades, as well as epochs with blinks or microsaccades that occurred at less than 200 ms after fixation onset. We focused on the early components at occipital electrodes O1 and O2, which are less prone to be affected by ocular artifacts. We then computed the positive and negative peaks in a predefined time range. The *P1* peak was measured using a 50–150 ms time range, and the *N1* was measured in a 100–200 ms window with no baseline correction. Finally, we calculated the baseline-corrected peak-to-peak *N1* relative to the *P1 magnitude* (*N1-P1*). Peak extraction was optimized by setting an individual time range for each observer at around their average peak latency, within the predefined time range, from all the conditions combined. This was done to avoid using a long time-range to overcome the latency differences across observers, which would increase the false peak discoveries. Extreme value artefacts were not allowed using a peak magnitude threshold exceeding ± 50 *µVolts*. To ensure that we used a similar number of epochs per participant, we used an estimation of an average of 3 saccades per second to include only the first 12 epochs per trial (trial duration = 4 s), in the final analysis.

### Statistical assessment

Usually, the statistical analysis of the variance (*One-way ANOVA*) and the Tukey multiple comparisons post-hoc tests were performed using Matlab. We first verified that the measured value distributions of different conditions come from normal distributions with equal variance. Another statistical method that was used is the *Linear Mix Model* (*LMM*)^51^. The responses were fitted to a simple model of maximum likelihood with the *serial saccade number,* or the *saccade size* used as the predictor variable, and the observer’s variability was set as the random effect. In addition, we computed Pearson's linear correlation coefficient (*r*^*2*^) for the group averages of each plot.

### Stimuli and procedure

**Figure 1:**
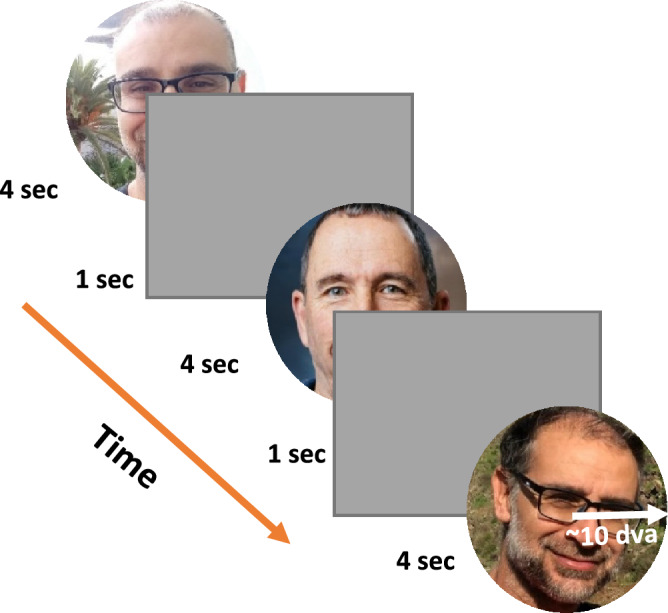
Stimuli and procedure. Observers freely inspected large facial images, presented in random order for four *seconds* each. In total, eight different identities were chosen of seven unfamiliar and one familiar world leader with five different images per identity. Note: the above images are of the authors who gave an informed consent to publish the images in an online open-access publication. They are used here for illustration only and were not used in the experiment.

Observers freely watched a slideshow of seven unfamiliar and one familiar world leader’s facial images presented randomly for four-*second* periods, with multiple different images per person (see Fig. 1). All images were chromatic with 600 × 800 minimum resolution, taken from the internet and were radially cropped around the head with a radius of 10 *dva*. The use of 5 different images per identity was done to reduce the effects of low image attributes and facial expressions from a specific image. In addition, we verified that the familiar face images were not appreciably different from the non-familiar face images regarding the low-level properties of luminance and RMS contrast. A task was not used, and the observers were just instructed to freely inspect the images.

## Results

We used static displays of faces, presented for four-second periods, while the observer viewed them freely without a task (see Fig. 1, “Stimuli and procedure”). Fixation-related potentials (FRPs), triggered by saccade landing time, were calculated, with a primary focus on early occipital components as well as Oculomotor-Inhibition (OMI), computed here as the fixation duration (MS-free fixations only) or the timing of the first microsaccade following the fixation onset, excluding corrective microsaccades occurring proximate to the preceding saccade landing (see the “Methods”).

### EEG-FRP results

**Figure 2:**
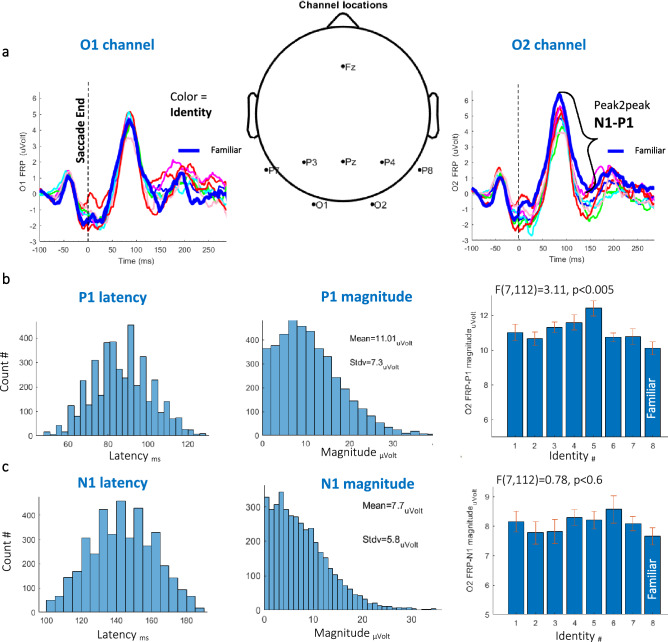
Basic FRP results. (**a**) Scalp EEG channel topography and O1, O2 FRP results for all the unfamiliar faces (thin lines) and one familiar face (the thick blue line) averaged across observers (*N* = 15). The vertical dashed line denotes the fixation onset. (**b**) Results for the O2-FRP *P1 peak latency* (left), magnitude (center), and P1 averaged across observers’ magnitudes for each of the identities (right) with error bars calculated over the demeaned data showing a significantly smaller magnitude for the familiar identities (*F*(7112) = 3.11, *p* < 0.005, *One-way ANOVA*). (**c**) Results for O2-FRP *N1 peak latency* (left), magnitude (center), and N1 averaged across observers’ magnitudes for each of the identities (right) with error bars calculated over the demeaned data showing a non-significant smaller magnitude for the familiar identities (*p* < 0.6).

The main familiarity effect was found at the posterior right hemisphere via the early occipital responses measured at the O2 electrode (see Fig. 2a for a view of the EEG channel locations). The FRP waveforms in Fig. 2a (left and right panels) were baseline corrected to the mean value of the data points preceding the fixation-inducing saccade spike potential (− 100 to − 75 ms). Then, we computed the peak magnitude (see the “Methods”) for the Lambda response (*P1*) peaking around ~ 90 ms*,* and the *N1* peaking around ~ 140 ms after fixation onset (see Fig. 2b,c). We then computed a baseline-corrected *N1* by subtracting the *P1 magnitude* (peak-to-peak) per fixation-related epoch (see the “Methods”), which allowed us to test the combined FRP *N1* and the *P1* response. We hypothesized that the *N1*, which is often associated with a bottom-up prediction-error (PE), would reflect less PE for familiar faces with smaller magnitudes due to prior visual knowledge, which facilitates oculomotor dynamics and visual processing.

### The O2 FRP N1-P1 familiarity effect

**Figure 3:**
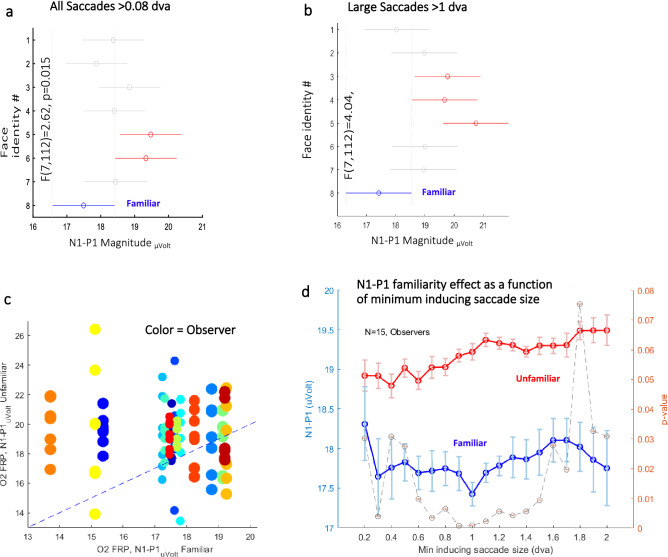
The baseline corrected N1 familiarity effect. (**a**) *N1-P1 magnitude* for each of the 8 identities averaged across observers with error bars calculated over the demeaned data using epochs triggered by saccades with sizes ranging from 0.08 to 20 *dva*. The results show a significantly smaller magnitude for the familiar identity (*p* < 0.015, *One-way ANOVA*) with post-hoc multiple comparisons tests yielding two groups that significantly differ from the familiar identity, with 95% *confidence intervals*. (**b**) The same as (**a**) but with saccade sizes ranging from 1 to 20 *dva.* The results show a smaller magnitude for the familiar identity (*p* < 0.0005, *One-way ANOVA*), with three groups that significantly differ from the familiar identity (post-hoc tests). (**c**) A detailed observer scatter plot with a different color per participant and 7 dots showing the unfamiliar identities; the *N1-P1 magnitude* was compared with the familiar identity, showing that most of the dots are above the diagonal, signifying a larger magnitude for the unfamiliar identity. The dot size was reduced in crowded areas for better visibility. (**d**) The *FRP N1-P1* familiarity effect (familiar denoted in blue and all the unfamiliar combined in red), was tested (Paired t-test) by systematically changing the threshold for the minimum saccade size taken as a trigger for the FRP. The p-values are plotted in light gray.

The baseline-corrected *N1 (N1-P1)* was calculated relative to the *P1 magnitude* for each epoch, with a mean value of *18.8 µVolt* ± *9 SD.* The *N1-P1* magnitudes were calculated for all conditions (8 different face identities) averaged across observers (*N* = 15) with error bars calculated over the demeaned data. We examined the importance of defining a threshold discriminating between fixation-inducing saccades and smaller microsaccades. We tested the familiarity effect by taking all saccade sizes (> 0.08 dva) as triggers for FRP. The results are shown in Fig. 3a. A significantly smaller *N1-P1 magnitude* was found for the familiar identity, compared with each of the unfamiliar identities, *p* < 0.015 (*F*(7112) = 2.62, *One-way ANOVA*). This effect was much smaller than the effect induced by larger (> 1 *dva*) saccades (*p* < 0.0005 (*F*(7112) = 4.04, *One-way ANOVA*, see Fig. 3b). A multiple comparisons test yielded three out of seven significantly different groups from the familiar identity, with an illustration of the *confidence intervals*. To account for the individual contribution to the results, a detailed observer *scatter plot* with a different color for each participant and a dot for an unfamiliar identity *N1-P1 magnitude,* compared with the familiar one, indicated that most of the dots are above the diagonal, signifying a larger magnitude for the unfamiliar one (see Fig. 3c).

Finally, we investigated the effect of systematically changing the threshold for the minimum size of the fixation-inducing saccades, on the FRP *N1-P1* from 0.2 to 2 *dva*. The results appear in Fig. 3d. Importantly, we found that the *N1-P1* value was not specific to the 1 *dva* threshold for the inducing saccade size, and it changed gradually, with the strongest effect (*p-value*) found in the range of 0.7–1.4 *dva*. Note that increasing the threshold also results in a reduced number of epochs and statistical power, whereas decreasing the threshold may add epochs induced by corrective saccades, which by themselves, do not produce a familiarity effect (e.g. 0.08–0.5 *dva*). The 1 *dva* threshold seems optimal, although the results remain robust and are in a much wider range.

### Adaptation effect on the FRP

**Figure 4:**
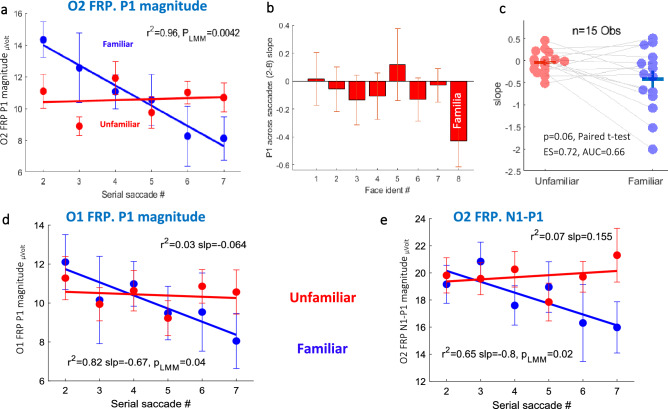
P1 adaptation effect. (**a**) *O2 FRP P1 magnitude* as a function of the *serial saccade number* showing an adaptation effect across the first seven saccades, for the familiar (*p* = 0.0042, *Linear Mixed Model*) but not for the unfamiliar identities combined. (**b**) Slopes of the *O2 FRP P1 magnitude* linear fit across successive saccades for each of the identities. (**c**) A *paired t test* comparison between the familiar identities and the combined unfamiliar *P1 slopes* showing an almost significant difference (*p* = 0.062). (**d**) *O1 FRP P1 magnitude* as a function of the *serial saccade number* showing an adaptation effect across the first seven saccades, for the familiar (*p* = 0.04, *Linear Mixed Model*) but not for the unfamiliar identities combined. (**e**) *O2 FRP N1-P1 magnitude* as a function of the *serial saccade number* showing an adaptation effect across the first seven saccades, for the familiar (*p* = 0.02, *Linear Mixed Model*) but not for the unfamiliar identities combined. In (**a**), (**d**) and (**e**), the data were averaged on observers and error bars were computed on the demeaned data.

We tested whether the adaptation of the occipital activity over successive saccades affects the FRP signal differently for the two categories. We found that the average *P1-magnitude* as a function of the *serial saccade number* was attenuated across successive saccades for the familiar identity via the O1 and O2 electrodes (*p* = 0.04, *p* = 0.0042, respectively. *Linear Mixed Model*), but not for the unfamiliar identity, with all identities combined (Fig. 4a and Fig. 4d). We then calculated the P1 (via O2) linear fit slopes separately for each of the identities and found that the average across observer negative slope was the largest for the familiar identity (Fig. 4b). A *paired t test* comparison was performed between the familiar slope values and the combined unfamiliar ones, showing nearly a significant difference (*p* = 0.06; see Fig. 4c), with a calculated medium effect size (*ES* = 0.72, *Cohen’s d*) and the area under the *ROC* curve (*AUC* = 0.66). The *baseline-corrected N1* also shows a similar adaptation for the familiar identity (Fig. 4e) as expected, because it was calculated relative to P1, peak-to-peak.

### The Fixation-related OMI familiarity effect

A recent study by Rosenzweig & Bonneh^23^ showed prolonged OMI for familiar faces briefly presented and masked. We wanted to determine whether microsaccade latencies after a saccade are also increased when a famous face is freely inspected for several seconds relative to an unfamiliar one.

**Figure 5:**
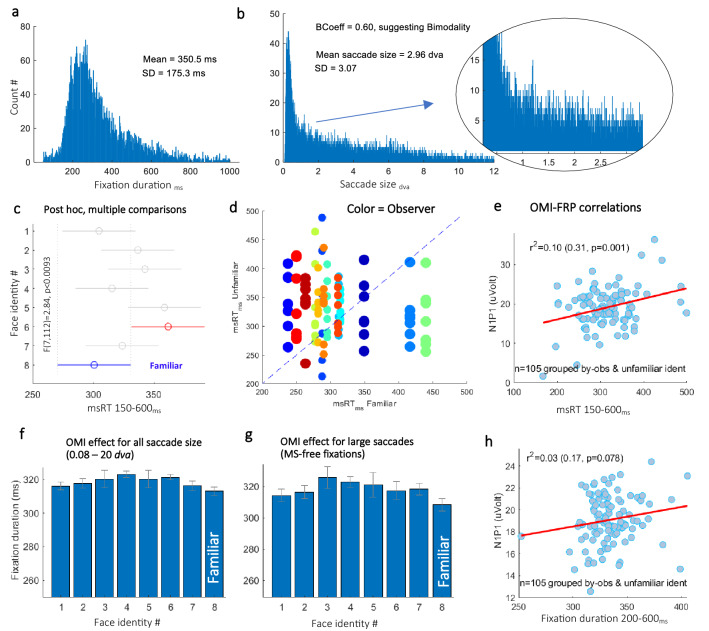
The Oculomotor Inhibition (OMI) familiarity effect. (**a**) A fixation duration histogram with a mean duration of 350.5 ms ± 175.3 *SD*. (**b**) A saccade size histogram (left) with a mean size of 2.96 *dva* ± 3 *SD* and a *Bimodality-Coefficient* of 0.6, suggesting that the saccade size have bimodality distributions. Magnification of the region between 0 and 2.5 *dva* reveals a second peak (right). (**c**) The OMI (msRT 150–600 ms) results for each of the 8 different face identities averaged across observers (*N* = *15*) showing a significantly shorter msRT for the familiar (*p* < 0.0093, *One-way ANOVA, 95% confidence-intervals*). (**d**) A detailed *scatter plot* with a different color per participant and a dot for an unfamiliar identity’s msRT, compared with the familiar. Note that most of the dots are above the diagonal, signifying a longer OMI for the unfamiliar identity. The dot size was reduced in crowded areas for better visibility. (**e**) A scatter plot with a dot for an unfamiliar identity; each of the observers showed a positive correlation (R = 0.31, p = 0.001, Pearson correlation) between the msRT and N1-P1 magnitude after demeaning within observer (see the “Results”). (**f,g**) The fixation duration after large saccades (> 0.08 *dva* in (**f**) and > 1 *dva* in (**g**)), for each of the 8 identities, averaged across observers using a 200–600 ms duration range and MS-free fixations only in (**g**). Like msRT, the fixation duration is shorter for the familiar, but the results were insignificant. (**h**) The same as in (**e**) but for fixation duration. The results show a nonsignificant relation (R = 0.17, p = 0.078).

The results for the OMI are shown in Fig. 5. Fixation duration as well as the saccade size histograms for all observers and conditions combined are shown in Fig. 5a,b. The mean fixation duration was 350 ms ± 175 *SD*. The saccade size distribution reflects a bimodal distribution of saccades and microsaccades (see Fig. 5b, right panel) with a calculation of the *bimodality coefficient* yielding a significance, *BC* = 0.6, suggesting bimodality (see the “Methods”). The mean microsaccade size was 0.42 *dva* ± 0.24 *SD*. We computed the msRT (microsaccade reaction time, see the “Methods”) as the first microsaccade (< 0.6 *dva*) occurrence from 150 to 600 ms after the fixation onset, ignoring the corrective microsaccades that may occur immediately after the saccade. Figure 5c shows the average msRT across observers for each of the identities, showing decreased OMI for the familiar identity, which is opposite to the results obtained with flashed faces^23^. Significance was assessed using *one-way ANOVA*, *F*(7112) = 2.84 and *p* < 0.0093. The *post-hoc Tukey* method tests indicated that the familiar identity was significantly different only from one other unfamiliar group. The observer was denoted by color, in the *scatter plot* (Fig. 5d), with a dot per identity, compared with the familiar identity. Interestingly, it was shown that most observers had longer msRTs for the unfamiliar identity (the dots above the symmetry line).

We found that the distinction between small and larger saccades was also critical for the OMI effect. When examining the intervals between all saccades (the fixation duration) with sizes from 0.08 and from 1 *dva* (Fig. 5f,g, respectively), the familiar had a shorter duration; however, there was no significant familiarity effect on the fixation duration. Finally, we examined the possible correlation between the FRP and OMI measures (Fig. 5e,h). To control for irrelevant factors affecting the *N1-P1* and OMI values, the data were demeaned within observers and within saccade size bins (1 *dva*). Then the grand average from all observers and saccade sizes was added. For this analysis we also excluded fixations triggered by saccade sizes > 8 *dva*. The msRT/fixation-duration and the *N1-P1*, grouped by unfamiliar identity and each of the observers, show a positive correlation (*R* = *0.31, p* = *0.001, and R* = *0.17*, *p* = *0.078*, Pearson’s correlation).

### The effect of saccade size

**Figure 6:**
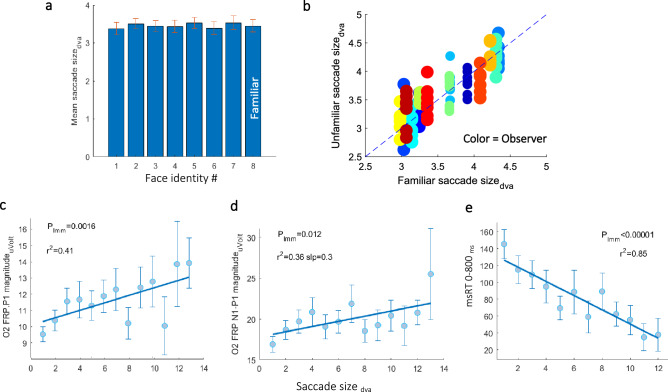
Saccade size effect. (**a**) Mean *saccade size* per identity, showing no difference between the familiar and unfamiliar identities. (**b**) A detailed observer scatter plot comparing the average saccade size for the familiar identities with each of the unfamiliar ones, for each observer (a different color). As shown, *saccade size* did not differ with familiarity, indicated by the balanced distribution of the dots along the diagonal line. (**c**) *P1-magnitudes* as a function of inducing the *saccade size,* averaged across observers, showing a significant positive relation (*p* = 0.0016, *LMM*, see the “Methods”). (**d**) FRP *N1-P1 magnitude* was also positively correlated with *saccade size* (*r*^*2*^ = 0.36, *Pearson correlation; p* = 0.012, *LMM*). (**e**) Microsaccade latency (msRT) shows a negative correlation with the *saccade size* (*r*^*2*^ = 0.85, *Pearson correlation; p* = 0.00001, *LMM*).

Previous FRP studies reported that the Lambda response (P1) amplitude increases with larger saccades^1,52,53^. This suggests that our finding of a decreased *N1-P1* peak-to-peak magnitude for the familiar identity could result from smaller saccades that reduce the P1 amplitude. We therefore examined the effect of *saccade size* on the FRP. Figure 6 shows the effect of inducing the *saccade size* on the FRP and the relationship between the saccade size and familiarity. The saccade size (> 1 *dva*) did not differ between familiar and unfamiliar identities when averaged across observers (Fig. 6a), or when plotted for each observer in a scatter plot (Fig. 6b). A significant positive relation (*r*^*2*^ = 0.41, *Pearson correlation*) of the *P1 magnitude* and the *saccade size* (*p* = 0.0016, *LMM*) is plotted in Fig. 6c, which is consistent with previous studies. Figure 6d shows that the *N1-P1 magnitude* was also positively correlated with saccade size (*r*^*2*^ = 0.36, *Pearson correlation; p* = 0.012, *LMM*), because it was calculated relative to the *P1 magnitude* (peak-to-peak). Finally, the corrective microsaccade latencies show a negative correlation with *saccade size* (*r*^*2*^ = 0.85, *Pearson correlation; p* = 0.00001, *LMM*); thus, larger saccades induced faster microsaccade reaction times due to the lower peripheral preview acuity (see Fig. 6e).

## Discussion

We investigated how familiarity in passive free viewing affected the early occipital responses that occur prior to face identification, and the effect on the latency of microsaccades, which is known to be affected by attention^46,54,55^ and expectations^18,56,57^. Our results revealed a smaller fixation-related *N1-P1 magnitude* and a shorter microsaccade inhibition (OMI) for the familiar faces, suggesting a lower prediction error while freely viewing familiar faces. Interestingly, the *N1-P1* FRP effect of familiarity appeared only at the right hemisphere, which is more specialized, according to some studies on face processing^58,59^.

We also examined the possible effect of adaptation and saccade size on the FRP and found that whereas saccade size did not differ between conditions and thus affected the FRP for different categories in the same way, the *P1 magnitude* was consistently adapted for the familiar faces and might have significantly contributed to the FRP familiarity effect. Next, we will discuss the three main factors that could affect the FRP results: (1) Prediction Error, PE, (2) Adaptation, and (3) Saccade size.

### With the help of prior knowledge: predictability/priming across saccades

We propose here that long-term familiarity provides a type of priming for the viewed image across saccades, revealed by a shorter OMI (see Fig. 5) and smaller occipital P1 and N1 amplitudes (see Fig. 3). These effects could be attributed to the effect of prior knowledge or long-term (over several seconds) priming by exposure to different photos having the same identity. Rare visual or auditory events are known to induce a longer OMI. For example, prolonged microsaccade inhibition has been reported for oddballs in a sequence, a rare blue patch among frequent red patches^18^, and for auditory deviants^57,60,61^. More preliminary evidence of prolonged inhibition was found for high-contrast patches among low-contrast patches^62^ and for temporal oddballs via unpredicted intervals^11,12^. The ERP Mismatch-Negativity (MMN) is a well-known electrophysiological marker for oddball response. It reflects an automatic change detection mechanism in the auditory domain^63^,Brain Generators Implicated in the Processing of Auditory Stimulus Deviance: A Topographic Event‐Related Potential Study, 1990^64,65^; it is manifested by a larger N1 negativity, peaking at about 170 ms at the temporal electrodes. Some studies suggest that a correlate of this prediction error generator also exists in the visual domain for visual mismatches termed vMMN^66^. Infrequent color patterns^67^, low spatial frequency gratings^68^, and face and house deviant orientations^69^ elicited a posterior vMMN. Even facial expressions and emotions were used in an oddball paradigm and elicited vMMN peaking at 100–200 ms^70–72^.

All the above are examples of larger PE, measured by longer OMI and larger N1 amplitudes. Priming by repetition, on the other hand, has an effect that is opposite that of PE, manifested by attenuated responses similar to adaptation, and behavioral facilitation. Previous N170 ERP studies of face familiarity reported priming effects via smaller N170 amplitudes. For example, a study that used moony faces and priming by the same photo or a different photo, but with the same identity, found attenuated N170 amplitudes for the familiar identity, suggesting a top-down feedback effect, whereas a stronger priming effect was found with the same photo^42^. Short-term, within a second, priming effects, indicated by smaller N170 amplitudes, were reported in a face memory recall task for repeated stimuli, using inverted and contrast-reversed unknown faces^44^. Another study found identity-specific priming effects via attenuated P1 and N170 using morphed faces and argued that they may be due to low-level visual similarities^73^. A very recent study by Buonocore, Dimigen et al. found a reduction of the fixation-related N170, following an extra-foveal face preview, and contended that it is due to prediction^5^.

In our recent paper, fixation-related OMI was measured for low-level stimuli in free viewing^19^. We found a resembling effect for OMI across saccades in the form of shortening of the OMI by repetition priming. Although this facilitation effect was found for gratings with the same spatial frequency, we believe that eye movement enhancement may occur due to familiarity or because different images have the same identity. Finally, we suggest that the N1 and OMI priming effects we found for the familiar identify did not occur independent of adaptation.

### Adaptation of the occipital P1 across saccades

Our results indicated that the occipital P1 for the familiar identity was attenuated across saccades (Fig. 4). At least half of the participants showed a larger P1 adaptation for the familiar than for the unfamiliar identity. This attenuation could be interpreted as a simple reduction of attention for the familiar identity, because P1 reflects low-level features activity; this could also explain the shorter saccadic inhibition because attended stimuli induce longer OMI. However, it could also be related to prediction and priming. Adaptation and priming are difficult to separate; they can co-exist in the current settings and discriminating between the two requires further research. The process of attenuation over successive saccades is reminiscent of the repetition suppression phenomenon, which was found for face category and identity via fMRI and EEG. Image-invariant adaptation for familiar faces, but not for unfamiliar faces, was found in the face-selective regions of the medial temporal lobe, MTL^74^. An fMRI study found a repetition suppression effect only for famous faces, whereas the opposite was found for unfamiliar ones (R.^75^. More ERP studies found N170 adaptation to face category^76^ as well as to face identity^77^. A later identity-specific adaptation on the ERP was also found over superior occipito-temporal sites at around 200-280 ms^78^.

There is a known asymmetry of the ventral visual cortex for face processing in the literature. It is evident from lesions to the left/right hemispheres that affect word or face processing^58,59^. In addition, the N170 also shows lateralization and is more prominent in the right hemisphere for faces than are words^3,79^. In the current study, adaptation of the P1 FRP for the familiar identity appeared on both the O1 and O2 electrodes, placed at the posterior sites of the two hemispheres (see Fig. 4a,d). However, the *N1-P1* FRP effect was found only through O2 (see Fig. 3). This can indicate that adaptation is not the main factor for the *N1-P1* FRP effect.

### The effect of saccade size on the FRP

In our study we found that the occipital *P1 magnitude* increased as a function of the amplitude of the saccade that precedes the fixation (see Fig. 6) as previously reported^1,52,53^. This suggests that the effect of decreased *N1-P1 magnitude* for the familiar identity could result from differences in the saccade amplitudes for different identities because the *N1-P1* negative peak value was baseline corrected by the preceding *P1* peak value, peak-to-peak (see the “Methods”). For this reason, we measured the saccade size for each identity and found no significant difference between different identities (Fig. 6a,b). The results also showed that the microsaccade timing after the saccade had a negative relationship with saccade size, meaning that after a large saccade, the corrective saccade was more immediate. In our OMI measurements we ignored the corrective saccades by including only microsaccade timings over 150 ms post-fixation onset.

### The distinction between larger saccades and microsaccades

Recent studies provide evidence that saccades and microsaccades share a common neurophysiological basis^10^ and perform similar functions^80^. This raises the question of whether the distinction we make between saccades with sizes > 1 *dva* as triggers for fixation-related responses is indeed important and whether the threshold we use is critical for generating the familiarity effect. We first noted that the main theme in the current study as well as in our previous study^19^ is that each saccade generates a transient stimulus to the visual system, such as the flashed stimuli in the ERP and OMI studies. Microsaccades also generate a transient visual stimulation, but their magnitude is smaller (see Fig. 6d,e, assuming that the occipital FRP magnitude will become smaller below 1 *dva,* not shown). The use of a 1 *dva* threshold in the current study was initially derived from a popular definition of microsaccades (e.g.^81–83^) corresponding to the size of the foveola, although other studies use smaller thresholds, e.g. 0.5 dva^84,85^. Overall, when considering all saccades as fixation-inducing, the FRP familiarity effect was still significant but degraded (compare Fig. 3a,b), whereas the OMI effect became insignificant (Fig. 5f,g). See FRP & OMI familiarity effect pars in Results.

### Comparison with previous eye-movement based concealed information studies

Previous familiarity studies that used eye tracking measurements detected fewer fixations with longer fixation durations when a familiar face was viewed^28^ and fewer areas of interest on the face when the participants were instructed to conceal their knowledge of familiarity^26^. With a memory task, familiar faces were less explored perhaps because they are easier to remember^29^. In our recent study with different eye measurements, a longer OMI for the familiar was found for microsaccades and for blinks when face stimuli were briefly presented and masked^23^. Here, we also used images with one familiar identity out of eight, as in our recent study, but with a longer presentation duration. We took care of the unequal sampling of the familiar and the unfamiliar faces by comparing the results for the familiar face to each of the equally sampled unfamiliar faces separately, having a chance level of 1/8 (12.5%) to find a difference. We expected to find similar results of longer OMI for the familiar in free viewing, assuming that familiarity would raise associations, episodic-memories, and emotions, thus delaying the next saccade/microsaccade. However, we found the opposite from the expected, shorter saccadic inhibition for the familiar identities, which might be related, as discussed before, to scanning enhancement due to priming/adaptation and feedback from other areas associated with prior knowledge.

## Summary and conclusions

When observers free viewed a set of familiar and unfamiliar faces for a few seconds per face, the fixation-related potentials (FRPs) showed a decreased *N1-P1 magnitude* for the familiar faces at the right occipital electrode and a shorter OMI for the familiar, compared with unfamiliar identities, both indicative of a smaller prediction error. The *P1 magnitude* for the familiar identity had been suppressed across successive saccades, implying priming or adaptation. In the current study, adaptation of the *P1 magnitude* for the familiar identity appeared on both hemispheres; however, the decreased *N1-P1 magnitude* effect was observable only through the right occipital electrode, O2; this suggests that predictive structural face features, reflected by *N1* rather than adaptation of low-level features, reflected by *P1*, is the main factor driving this effect. Overall, the results indicate the sensitivity of the occipital FRP and the OMI in free viewing in relation to face familiarity; this could be used as a novel physiological measure for studying hidden memories.

## Data Availability

The experimental datasets generated during the current study will be available from the corresponding author upon reasonable request.
